# Thiosulphate sulfurtransferase: Biological roles and therapeutic potential

**DOI:** 10.1016/j.redox.2025.103595

**Published:** 2025-03-14

**Authors:** Yang Luo, Shaden Melhem, Martin Feelisch, Laurent Chatre, Nicholas M. Morton, Amalia M. Dolga, Harry van Goor

**Affiliations:** a University of Groningen, Dept. of Molecular Pharmacology, Groningen Research Institute of Pharmacy, Faculty of Science and Engineering, Groningen, the Netherlands; b University Medical Center Groningen, Dept. of Pathology and Medical Biology, Groningen, the Netherlands; c Centre for Cardiovascular Science, Queen's Medical Research Institute, University of Edinburgh, Edinburgh, UK; d Clinical and Experimental Sciences, Faculty of Medicine, University of Southampton and University Hospital Southampton NHS Foundation Trust, Southampton, UK; e Université de Caen Normandie, CNRS, Normandie Univ, ISTCT, UMR6030, GIP Cyceron, Caen, F-14000, France; f Centre for Systems Health and Integrated Metabolic Research, School of Science and Technology, Nottingham Trent University, Nottingham, UK

**Keywords:** Thiosulfate sulfurtransferase (TST), Mitochondrial dysfunction, Oxidative stress, Redox signaling

## Abstract

Mitochondria are central to eukaryotic cell function, driving energy production, intermediary metabolism, and cellular homeostasis. Dysregulation of mitochondrial function often results in oxidative stress, a hallmark of numerous diseases, underscoring the critical need for maintaining mitochondrial integrity. Among mitochondrial enzymes, thiosulfate sulfurtransferase (TST) has emerged as a key regulator of sulfur metabolism, redox balance, and Fe–S protein maintenance. Beyond its well-known role in cyanide detoxification, TST facilitates hydrogen sulfide (H_2_S) metabolism by catalyzing the transfer of sulfur from persulfides (R–SSH) to thiosulfate (S_2_O_3_^2−^), promoting H_2_S oxidation and preventing its toxic accumulation. Additionally, TST contributes to the thiol-dependent antioxidant system by regulating reactive sulfur species and sustaining mitochondrial functionality through its role in sulfide-driven bioenergetics.

This review highlights the biochemical and therapeutic significance of TST in mitochondrial and cellular health, emphasizing its protective roles in diseases associated with oxidative stress and mitochondrial dysfunction. Dysregulation of TST has been implicated in diverse pathologies, including specific metabolic disorders, neurological diseases, cardiovascular conditions, kidney dysfunction, inflammatory bowel disease, and cancer. These associations underline TST's potential as a biomarker and therapeutic target.

Therapeutic strategies to activate the TST pathway are explored, with a focus on sodium thiosulfate (STS), novel small molecule (Hit 2), and recombinant hTST protein. STS, an FDA-approved compound, has demonstrated antioxidant and anti-inflammatory effects across multiple preclinical models, mitigating oxidative damage and improving mitochondrial integrity. A slow-release oral formulation of STS is under development, offering promise for expanding its clinical applications. Small molecule activators like Hit 2 and hTST protein have shown efficacy in enhancing mitochondrial respiration and reducing oxidative stress, though both reagents need further *in vitro* and *in vivo* investigations.

Despite promising advancements, TST-based therapies remain underexplored. Future research should focus on leveraging TST's interplay with pathways like NRF2 signaling, investigating its broader protective roles in cellular health, and developing targeted interventions. Enhancing TST activity represents an innovative therapeutic approach for addressing mitochondrial dysfunction, oxidative stress, and their associated pathologies, offering new hope for the treatment of diseases associated with mitochondrial dysfunction.

## Highlights

• The mitochondrial interaction between oxidative stress and TST.
• The extra-mitochondrial impact of TST on NRF2 signaling.
• Tissue expression profile of TST in various diseases.
• Therapeutic potential of TST activation.

## Graphical abstract

**Figure:**
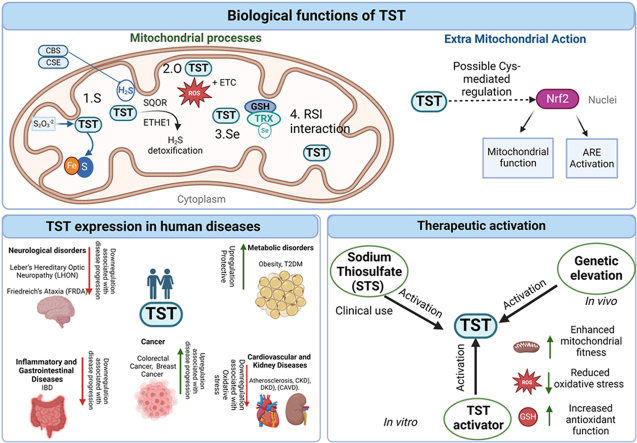

## 1 Introduction

With a few exceptions (e.g. erythrocytes), mitochondria are present in the majority of eukaryotic cells where they act as crucial integrators of cellular intermediary metabolism [1]. Secondary to oxidative phosphorylation (OXPHOS), mitochondria also play a critical role in amino acid and lipid metabolism, heme and iron–sulfur (Fe–S) cluster biosynthesis, calcium homeostasis and cell death pathways [[2], [3], [4], [5]]. In diseases with mitochondrial dysfunction, the dysregulated metabolism leads to excessive formation of reactive oxygen species (ROS) such as superoxide (O_2_^•−^) and hydrogen peroxide (H_2_O_2_). Under conditions of impaired scavenging, ROS accumulation leads to oxidative stress [6]. This exacerbates mitochondrial dysfunction, creating a vicious cycle where mitochondrial impairment leads to further increased ROS production and aggravated oxidative stress [7]. Protecting the structure and function of mitochondria is therefore critical to the integrity of cellular activity and preservation of organ function.

Mitochondrial diseases (MDs) are a class of hereditary disorders that are characterized by malfunctioning mitochondria caused by mutations in either nuclear or mitochondrial DNA (mtDNA) [8]. Common MDs (distinguished as childhood- or adult-onset MDs) include clinical syndromes such as Leigh syndrome, Alpers–Huttenlocher syndrome (AHS), Leber hereditary optic neuropathy (LHON) and Kearns–Sayre syndrome (KSS) [8]. Besides these genetic MDs, there are secondary conditions where an acquired mitochondrial dysfunction contributes to disease processes in the absence of a primary genetic mutation. Diseases with mitochondrial dysfunction are clinically heterogeneous, can occur at any age and can manifest with a wide range of clinical symptoms [9,10]. Their pathological outcome can impact any organ or tissue, but typically affects organs that are highly dependent on aerobic metabolism, such as the central nervous system, skeletal and cardiac muscles, kidneys, liver, and endocrine systems [8,11].

Among the hundreds of mitochondrial enzymes in existence, thiosulfate sulfurtransferase (TST, EC 2.8.1.1; also known as rhodanese), is particularly highly expressed in organs with high metabolic demand such as liver, colon, kidney, and brain [12]. The sulfurtransferase (ST) family comprises a diverse group of enzymes, primarily differentiated by the presence of single or multiple alpha/beta domains [[12], [13], [14]]. Structurally, TST enzymes exhibit either a single rhodanese domain, such as human TSTD1 and bacterial GlpE and PspE, or a tandem repeat of rhodanese domains, as observed in human TST (hTST), Bos taurus (Rhobov), and Azotobacter vinelandii (RhdA) [12,15]. The 3D structure of Rhobov (PDB 1BOH) has been well characterized, providing insight into the structural organization of this family. The three-dimensional structure of human TST, while not experimentally solved, has recently been predicted using AlphaFold, and is publicly available under the identification code AF-Q16762-F1-v4 (https://alphafold.ebi.ac.uk/entry/Q16762). The gene encoding this enzyme is located on chromosome 22 (22q12.3), and the protein itself is composed of 297 amino acids with a molecular weight of 35.6 kDa [14]. A wealth of literature exists describing the details of the catalytic activity and structural characteristics of TST [12,16]. We here discuss the biological pathways/proteins with known TST-mediated modulation across various experimental models and diseases, since they offer valuable insight into their potential therapeutic roles in diseases associated with mitochondrial dysfunction, given the widespread pathological phenotypes these diseases share.

## 2 Biological role of TST in mitochondrial processes

Mitochondria, often referred to as the “powerhouse of the cell”, exhibit remarkable plasticity and dynamism, enabling cells to adapt and respond to various environmental stressors and metabolic demands [1]. Being the location of crucial biochemical processes such as fatty acid oxidation (FAO), oxidative phosphorylation (OXPHOS), reactive species production, redox balance etc., it is perhaps not surprising that many studies into mitochondrial protein functions have been conducted [17,18]. Mitochondrial TST was initially discovered in 1933 as a cyanide (CN^−^) detoxifying enzyme that forms non-toxic thiocyanate (SCN^−^; also called rhodanide) by using thiosulfate (S_2_O_3_^2−^) as a sulfur donor [19]. Excessive cyanide can be highly toxic by binding to the ferric ion (Fe^3+^) of complex IV, thereby inhibiting oxidative metabolism and ultimately causing cell death [20,21]. This enzymatic process forms the basis for the use of thiosulfate as an antidote to cyanide poisoning. 3-mercaptopyruvate sulfurtransferase (MPST, EC 2.8.1.2), another enzyme with known rhodanese activity, is evolutionarily believed to be closely linked to TST because of its high sequence homology. Both TST and MPST belong to the rhodanese/Cdc25 phosphatase superfamily [22]. TST is present in the mitochondria while MPST is localized in both cytosol and mitochondria of eukaryotic cells [23,24]. Besides cyanide detoxification, TST is involved in a wealth of additional beneficial roles across various organs [25]. For the benefit of future pharmaceutical development of chemical entities capable of activating TST, we summarize key sights from TST signaling and propose various possibilities that appear to have potential for the treatment of MDs.

### 2.1 Sulfur metabolism

Sulfur is an essential element for all known forms of life. Eukaryotic organisms lack the ability to reduced sulfate and thus have to take it up in the form of sulfur-containing amino acids (from protein). In cells, sulfur exists in a variety of oxidation states ranging from S^2−^ (sulfide) to S^6+^ (sulfate) [[26], [27], [28]]. The stable and labile forms of sulfur together compromise the sulfur pool [29]. The stable forms include the two proteinogenic amino acids, l-cysteine (Cys) and l-methionine (Met), and the labile forms are divided into sulfane sulfur (S^0^) and acid-labile sulfur including compounds such as persulfides, polysulfides and thiosulfate [30]. TST plays a key role in sulfur metabolism by facilitating the transfer of sulfur atoms between mobile small molecules and cellular proteins including the interaction with iron-sulfur (Fe–S) proteins, which will impact other pathways involved in sulfur metabolism, such as the interaction with hydrogen sulfide (H_2_S) signaling, antioxidant and Fe–S proteins [30].

#### 2.1.1 H_2_S metabolism

TST has been the subject of detailed investigation in the context of sulfide detoxification. H_2_S can be released from a labile sulfur pool, and was firstly identified in 1942 in mammalian tissue homogenates by the American biochemist Vincent Du Vigeneaud [31]. More recently, the pioneering work of Abe and Kimura confirmed the role of H_2_S as a gaseous neuromodulator and vasorelaxant in conjunction with two other physiological signaling molecules, i.e. carbon monoxide (CO) and nitric oxide (NO) [32,33]. In high concentrations, H_2_S is widely recognized for its toxic effects and has been implicated in fatalities among agricultural and industrial workers. Its toxicity primarily arises from the reversible inhibition of cytochrome C oxidase (complex IV), the final complex in the mitochondrial electron transport chain [34]. Over the past decade, significant research has been dedicated to elucidating the biological role of H_2_S in health and disease and exploring its potential therapeutic applications [35]. Two essential cytosolic H_2_S-producing enzymes are cystathionine-β-synthase (CBS, EC 4.2.1.22) and cystathionine-γ-lyase (CSE, EC 4.4.1.1). These enzymes catalyze the pyridoxal 5′-phosphate-dependent conversion of homocysteine to cystathionine and cystathionine to cysteine, respectively, resulting in the production of H_2_S (and glutathione; see below) [35]. Interestingly, targeted knock down of CBS/CSE/MPST in mice revealed that CBS, CSE and MPST are not the major sources of sulfide and eventually persulfide production. The authors show the involvement of cysteinyl-tRNA synthetase (CARS) as cysteine persulfide synthase (CPERS) in the biosynthesis of persulfides and in sulfur metabolism as opposed to the role of the three canonical sulfide/persulfide-generating enzymes (CBS/CSE/MPST) [36]. Furthermore, mammals harbor the enzyme MPST (located mainly in mitochondria), which forms a reaction with cysteine to create H_2_S in conjunction with cysteine aminotransferase (CAT) [37]. Accumulating evidence has demonstrated the importance of TST in H_2_S metabolism with sulfide quinone oxidoreductase (SQOR) and persulfide dioxygenase (ETHE1/PDO) to maintain sulfur homeostasis [38]. Global TST gene silencing in mice displayed an apparently diabetogenic phenotype, the circulating sulfide showed a 13-fold and 10-fold elevation in plasma and blood, while thiosulfate, the main oxidative metabolite of H_2_S, showed a 20-fold and a 475-fold increase in plasma and urine compared to healthy wildtype controls [[39], [40], [41], [42]]. However, in the cerebral cortex *Tst*^*−/−*^ mice displayed similar steady-state levels of H_2_S and thiosulfate when compared to controls, as observed in the liver [39,43]. The liver serves as the primary organ for H_2_S detoxification. In *Tst*^*−/−*^ mice, MPST protein levels dramatically dropped in the brain cortex and mitochondria, but despite lower mRNA for Mpst, protein levels were raised in the liver of *Tst*^*−/−*^ mice, possibly to compensatorily enhance sulfide elimination [39,43]. Remarkably, the deletion of TST did not affect the protein expression of CBS and CSE in murine liver, suggesting that elevated circulating sulfide and thiosulfate level are a result of impaired sulfide oxidation and reduced thiosulfate utilization [39]. Although TST also contributes to H_2_S formation by using dihydrolipoic acid (DHLA), its primary role is H_2_S catabolism [[44], [45], [46]].

#### 2.1.2 A thiol-dependent antioxidant interactome

For decades, an imbalance in the formation of pro-oxidant and antioxidant species favoring the former has been the classical definition of ‘oxidative stress’ [47]. A number of experimental observations led to the redefinition of oxidative stress as a condition linked to changes in redox signaling and control [48]. This triggered an updated interpretation of the original concept separating physiological oxidative stress (known as "oxidative eustress") from excessive and harmful oxidative stress (known as "oxidative distress"). Glutathione (GSH), a tripeptide composed of glutamate, cysteine and glycine, serves as one of the most abundant antioxidants in the cellular defense against oxidative stress. The reduction of ROS by GSH leads to the formation of glutathione disulfide (GSSG), and a low GSH/GSSG ratio is often used to be indicative of oxidative stress [49,50]. Human SQOR as well as TST can provide a sulfane sulfur to GSH, giving rise to the formation of glutathione persulfide (GSSH), a superior antioxidant compared to GSH which can even reduce thioredoxin (TXN; see below) [51]. Moreover, sulfane sulfurs can be directly fed into both antioxidant systems by TSTD1 and MPST [16,46,52]. GSSH can also be converted to thiosulfate (regenerating GSH) by TST utilizing sulfite [53]. Thus, in mammalian cells TST exhibits anti-oxidative functions by interacting with both the thioredoxin system and the GSH system. This notion is consistent with the aberrant GSH and GSSG content detected in the brain of *Tst*^−/−^ mice, where GSH was 36 % lower in *Tst*^−/−^ mice, and its oxidized form, GSSG, increased five times. As a result, the GSH/GSSG ratio declined 7.2 times in *Tst*^−/−^ mice, indicating an impaired redox balance due to the absence of TST enzyme activity. Moreover, in global *Tst*^−/−^ mice, GSH metabolism was found to be significantly reduced in the liver, whereas GSH levels were increased approximately 2-fold in the plasma for peripheral insulin sensitization [39,54]. Marutani et al. demonstrated that treating human neuroblastoma cells (SH-SY5Y) and murine primary cortical neurons with sodium thiosulfate, a substrate for TST and a donor of H_2_S, significantly elevated intracellular thiosulfate levels and moderately increased the concentration of GSH [55].

The thioredoxin (TXN) system, a disulfide reductase system, is another major antioxidant system in mammalian cells, maintaining a reducing environment by mediating the transfer of electrons from reduced nicotinamide adenine dinucleotide phosphate (NADPH) via TXN reductase to TXN, which subsequently uses highly conserved vicinal thiol groups to reduce its target proteins [56]. TXN2 is specifically localized in mitochondria, while TXN1 is primarily expressed in the cytosol; both proteins utilize NADPH as a cofactor for their activity [57]. TST degrades ROS with the help of TXN in cell-free systems and suppresses oxidative stress in the liver of animals exposed to radiation [[58], [59], [60], [61]]. Additionally, TST was reported to be able to regulate thioredoxin metabolism, via using TXN as a sulfur-acceptor substrate and acting as a TXN oxidase, verse vice, TXN2 also reduces propenylsulfur protein to restore TST activity in a dose- and time-dependent manner [[62], [63], [64], [65]]. The persulfide moiety (R–S–SH) of the covalently substituted rhodanese in the sulfurtransferase reaction (and an analogous sulfenic acid structure (R–S–OH) when the enzyme functions as a thioredoxin oxidase) account for these activities [62]. In the brain tissues of *Tst*^−/−^ mice, a decreased *Txn2* mRNA level was observed, while the *Txn1* mRNA level remained steady when compared to healthy control mice [66]. TXN may also be modulated through H_2_S-signaling [[67], [68], [69]]. However, the direct link existing between TXN, H_2_S signaling and TST activity has not been highlighted before.

#### 2.1.3 Iron-sulfur protein interactions with TST

Iron-sulfur (Fe–S) clusters make up the biggest class of metalloproteins in biology and are among the most frequent cofactors used by nature [70]. Fe–S clusters serve as versatile prosthetic groups in proteins that perform a range of tasks in living organisms, not only serving as cofactors in enzyme catalysis, but also assisting in Lewis acid reactions with mitochondrial aconitase and radical S-adenosylmethionine (SAM) enzymes [71,72]. Besides, these clusters also regulate gene expression in response to oxidative stress as well as changes in oxygen and iron levels [[73], [74], [75]]. Especially within mitochondria, Fe–S centers play vital roles in both the tricarboxylic acid cycle (TCA) and the electron transport chain (ETC). Fe–S centers transfer electrons donated by NADH and FADH_2_ in NADH dehydrogenase (Complex I), succinate dehydrogenase (Complex II) and cytochrome C - oxidoreductase (Complex III) of the ETC, with molecular oxygen serving as the final electron acceptor, forming water, at the level of Complex IV [70]. Unsurprisingly, enzymes containing Fe–S clusters are particularly susceptible to iron deficiency and oxidative stress [76,77], as the instability and gradual degradation of these clusters can cause irreversible damage to the enzyme's protein backbone [78]. Sulfurtransferases including MPST and TST are also involved in iron-sulfur protein clusters biogenesis and restoration. TST was reported to be directly participating in Fe–S cluster reconstitution and repair [79,80]. TST with endogenous thiosulfate as substrate are key prerequisites for the reconstitution of enzyme activity of Complex I, Complex II, NADH-nitrate reductase, and bacterial ferredoxin [79,[81], [82], [83]]. In the presence of ferric iron (Fe^3+^), TST and thiosulfate are able to restore the activity of spinach ferredoxin and Nitrogenase of *Klebsiella pneumoniae.* [84,85] Tangiguchi and Kimura et al. also discovered that MPST cooperates with 3-mercapyruvate and ferrous iron (Fe^2+^) to increase adrenal ferredoxin activity [86]. In line with the interactive function of TST, adipose tissue from Ad-Tst mice exhibited a higher protein level for Complex II and higher Complex II protein level was maintained with HFD in the Ad-Tst mice. By contrast, Fe–S containing mitochondrial aconitase (ACO2) and cytosolic ACO1 were similar during TST overexpression [87]. Those results showed the selective interaction of TST on different Fe–S proteins.

### 2.2 Oxygen metabolism

Molecular oxygen (O_2_) composes 21 % of the earth's atmosphere, which has become essential to almost all aerobic life forms for efficient energy (ATP) supply by mitochondria. In the mitochondrial ETC, oxygen takes up free electrons in a stepwise fashion while indirectly interacting with fatty acid oxidation. During this process ROS like O_2_^•-^ and hydroxyl radical (HO·) as well as the oxidant H_2_O_2_ are generated and can leak to the adjacent cellular environment [[88], [89], [90]]. Regardless of the importance of the ETC for cellular ATP production, the associated oxygen metabolites can be toxic. We are equipped with a highly effective system inasmuch as >95 % of the oxygen we breathe in is converted into H_2_O, but a small percentage also forms superoxide anion radicals (O_2_^•-^) [91]. In mitochondria, OXPHOS Complexes I and III are the primary generators of O_2_^•-^; due to its unstable nature, O_2_^•-^ will be swiftly converted by superoxide dismutase 1 (SOD1) and superoxide dismutase 2 (SOD2) into hydrogen peroxide (H_2_O_2_) [92,93]. These reactive intermediates can interact with other organic compounds such as proteins, lipids and nucleic acids, and lead to oxidative damage and cell death [90]. In section 2.1.2, we introduced the definition of oxidative distress, which occurs due to an inadequate antioxidant capacity to excessive ROS. As a result of losing TST's ability to interact with the GSH system, knockdown of *Tst* resulted in elevated mitochondrial ROS levels in 3T3-L1 adipocytes following exposure to oxidative stress induced by 1 % H_2_O_2_ [87]. Besides, ROS-sensitive adiponectin release from 3T3-L1 adipocytes was reduced by TST activity inhibition with 2-PTS treatment [87]. On the contrary, ROS production after 3T3-L1 cells were treated with Na-palmitate or hydrogen peroxide, thiosulfate supplementation can diminish mitochondrial ROS level, confirming TST's antioxidative significance [94]. In the cerebral cortical area of mice with global gene silencing of TST, the O_2_^•-^ level was 10 % higher in the cortex of *Tst*^−/−^ mice, while tissue H_2_O_2_ concentrations being 57 % higher in *Tst*^−/−^ mice compared to C57BL/6J control mice. Upon challenging these mice with paraquat (PQ, an oxidative stress inducer), *Tst*^*−/−*^ mice brain cortexes displayed a more deteriorated antioxidant system [66]. With treatment of yohimbine in HFD rats, lipid peroxidation decreased when TST expression was elevated [95]. The administration of hTST protein, as well as administration of the substrate of TST (thiosulfate) in zebrafish effectively mitigated oxidative damage induced by hyperglycemia [25]. Taken together, these results support the importance of TST for ROS scavenging and maintenance of redox balance.

As an upstream process of OXPHOS, fatty acid oxidation (FAO) indirectly interacts with oxygen molecules, and TST has been identified genetically as an obesity-resistance candidate in >60 generations of a polygenic “lean” mouse model. In a cohort of nearly 700 individuals from Iceland, an inverse correlation was found between *TST* mRNA levels in subcutaneous adipose tissue and body mass index (BMI). Furthermore, mice with transgenic Tst overexpression in mature adipocytes, showed resistance against HFD-induced obesity. Elevated *Tst* mRNA and protein expression in white adipose tissue provided protection against obesity-related pathologies [75]. These findings provided evidence of an interaction between TST and lipid metabolism. As further validation, *Tst* overexpression in adipocytes was correlated with an increase in the basal mRNA levels of liver carnitine palmitoyltransferase 1a (*Cpt1a*), which is important in long-chain FAO [87]. Zheng et al. observed positive associations between TSTD1/*Tstd1* and pathways related to cholesterol or lipid metabolism, via the modulation of high-density lipoprotein (HDL) levels in over 70 transcriptomic datasets [96]. The following studies showed primary hepatocytes from *Tst*^−/−^ mice exhibited an impaired medium-chain FAO stimulated by octanoate [39]. Thiosulfate augmented succinate metabolism represented by increased oxygen consumption rates in mitochondria [87].

### 2.3 Selenium metabolism

The trace element selenium (Se), the presence of which in our diet is crucial for health, mediates its actions through incorporation (as selenocysteine) into selenoproteins many of which exist in mitochondria. Twenty-five selenoprotein genes have been identified in the human genome including thioredoxin reductases (TXNRDs) and GPX4 [97,98]. These enzymes are vital for processes like the GSH-dependent detoxification of hydrogen peroxide and the provision of reducing equivalents to thioredoxin (TRX) and TXNRD system, enhancing cellular antioxidant defenses. TST plays a critical role in selenium metabolism by binding selenium in a 1:1 ratio, forming a stable perselenide (R-S-Se^-^) structure, as demonstrated *in vitro* [99]. This activity of TST is crucial for generating the reactive form of selenium needed to synthesize selenophosphate (SePO_3_), an essential donor for SeCys-tRNA, which is a precursor for selenocysteine [100]. Consequently, TST indirectly supports the synthesis of selenoenzymes. TSTD1 has been reported to be able to donate S_2_O_3_^2−^ to TRX, and alternatively TRX could potentially function as a persulfide donor [64]. On the other hand, the absence of TST induced the GPX4 protein expression to decrease in mice brain [66]. When excess free selenium accumulates to a toxic level, bovine liver rhodanese can tightly bind to selenium, although the binding seems to be ineffective for selenium delivery *in vivo* [99]. Based on these functions, selenoenzymes and by extension TST could exert antioxidant effects against cellular damage in e.g. inflammation, apoptosis and ferroptosis [98,101].

### 2.4 The reactive species interactome (RSI)

The reactive species interactome (RSI) is a recently defined conceptual framework that aims to integrate the interaction between the above discussed ROS with reactive nitrogen species (RNS such as NO, N_2_O_3_ and peroxynitrite, ONOO^−^), reactive sulfur species (RSS including hydrogen sulfide (H_2_S)-derived reactive persulfide and polysulfide species), and reactive carbonyl species (RCS such as the ferroptosis-related marker malondialdehyde, MDA) [102,103]. As the name ‘interactome’ implies, the RSI also includes redox enzymes such as superoxide dismutase (SOD), catalase, myeloperoxidase (MPO), TXN and GPX, and their downstream biological targets [102]. Importantly, the RSI is also tightly connected to cellular bioenergetics via its link to mitochondrial metabolism [103,104]. Given that the RSI extends well beyond ROS, a recent example includes the dysregulation of the RSI and OXPHOS remodeling through specific enhancing of complex IV activity by TST deficiency in the brain cortex [43]. TST deficiency lowered the RSS H_2_S and the RNS ONOO^−^, while it increased H_2_S_n_, and the ROS O_2_^−^ and H_2_O_2_. In addition, first-line antioxidant defense was affected by TST loss with specific lowering in SOD activity and GSH, increase in catalase activity and GSSG. Facing paraquat-mediated oxidative distress, TST loss further aggravated the antioxidant response through the dysregulation of the RSI in brain cortex [43]. Altogether, TST is involved in the RSI-mitochondrial axis, positing this protein as a major player in the emerging field of ‘redox medicine’ related to mitochondrial dysfunction and pathophysiological processes such as cancer, neurodegeneration and other mitochondrial diseases.

## 3 Extra-mitochondrial actions of TST related to NRF2 signaling

While many studies have demonstrated the antioxidant and respiratory functions of TST within mitochondria, there are few reports of how TST may affect cellular functions outside of this organelle. As the understanding of our bodily defense system evolves, there is an increased appreciation that the transcription factor NRF2 (nuclear factor erythroid 2-related factor 2) is of particular importance as master regulator that controls the expression of genes associated with antioxidant defense processes including GSH metabolism and mitochondrial function [105]. NRF2 regulation functions primarily at the protein level. Most existing research has primarily focused on the electrophile and redox sensor in Kelch-like ECH-associated protein 1 (KEAP1) and its role in modulating the NRF2 protein levels in response to metabolic changes [106].The interaction between KEAP1 and NRF2 is disrupted by electrophilic alteration or oxidation of cysteine thiols in KEAP1, allowing cells to respond to environmental stress. The ubiquitin E3 ligase activity of the KEAP1-CUL3 complex declines, and NRF2 is stabilized. The stabilized NRF2 accumulates in the nucleus and activates its target genes, which leads to an enhancement of antioxidant capacity [107]. Additionally, numerous studies have linked the TST-related antioxidant system to NRF2 signaling, mainly due to its transcriptional activation of GSH-related enzymes, which serves as an early defense against oxidative stress [[108], [109], [110], [111]]. Furthermore, H_2_S mediates direct persulfidation of KEAP1 and thereby contributes to sulfide-mediated NRF2 regulation [112].

Currently, the interaction between TST and NRF2 has been investigated in two studies. Protein levels of NRF2 appear to be lower and those of its intracellular inhibitor Keap1 higher in the brain of *Tst*^*−/−*^ mice. As a consequence of reduced NRF2 protein expression, the ARE genes including *Hmox1*, *Txn2*, *Gclc*, *Gclm* and *Gr* showed significant reduction in the mRNA levels in the absence of TST [43]. A similar decrease in NRF2 activation in *Tst*^*−/−*^ mice was observed in liver when assessed through transcription factor binding site (TFBS) enrichment analysis. In line with the diminished hepatic NRF2 activation, 10 of 47 known NRF2-regulated proteins were reduced in the liver of ND-fed *Tst*^*−/−*^ mice compared with C57BL/6J wildtype mice [39]. While different methods have been used in these studies, the decreased NRF2 expression and activation supports the notion that TST is important for normal NRF2 signaling.

**Fig. 1:**
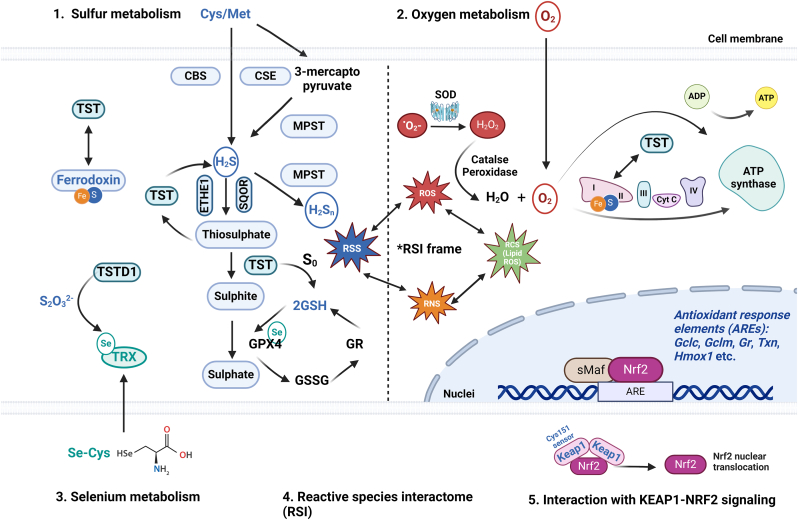
A conceptual framework of biological effects of TST in mammalian cells. 1. Sulfur metabolism: Sulfur is essential for redox signaling, H2S formation and antioxidant defense. TST plays a role in H2S metabolism, working with enzymes such as MPST. TST interacts with the thiol-dependent antioxidant system GSH and TXN systems, essential for cellular antioxidant defense. TST deficiency causes oxidative stress by disrupting redox balance, increasing ROS and lowering GSH levels. Additionally, TST supports the function of iron-sulfur (Fe–S) clusters, essential for mitochondrial processes like the electron transport chain (ETC). It helps to protect and restore Fe–S enzymes under oxidative stress conditions. 2. Oxygen metabolism: oxygen supports ATP production but also generates ROS, potentially damaging cells. TST interacts to regulate ROS level, maintaining redox balance. Studies indicate TST's antioxidative role extends to reducing mitochondrial ROS under conditions of oxidative stress. 3. Selenium metabolism: Selenium is crucial for the function of selenoproteins like GPX4 and TXN, which are essential for detoxifying excessive ROS. TSTD1 has been reported to be able to donate sulfane sulfur from S2O32− to TRX, and alternatively TRX could potentially function as a persulfide donor. 4. Reactive Species Interactome (RSI): The RSI integrates multiple reactive species, including ROS, RNS, RSS, and RCS, with enzymatic cellular antioxidant and redox pathways and mitochondrial activity. TST plays a significant role in maintaining redox balance. 5. Extra-mitochondrial TST functions and NRF2 signaling: TST impacts antioxidant responses by possible interaction with KEAP1 protein mediated by H2S, further influencing NRF2, a key regulator of genes that manage oxidative stress. NRF2 activation enhances antioxidant defenses, linking TST's roles both within and beyond mitochondria. Abbreviations are listed in Table 1.

Due to the complexity of the NRF2 system, the hypotheses related to the interaction between TST and NRF2 so far focussed mainly on H_2_S signaling. Numerous investigations have demonstrated that one mechanism by which H_2_S directly interacts with the NRF2 pathway is S-sulfhydration of cysteine-151 residue of Keap1 [111]. Furthermore, KEAP1 can be S-sulfhydrated by H_2_S at the cysteine-226 and cysteine-613 residues, which will inactivate KEAP1, release NRF2, and encourage the production of NRF2-dependent genes [113]. TST serves as modulator of sulfide metabolism, with circulating sulfide increasing dramatically in *Tst*^*−/−*^ mice, which might explain the disrupted NRF2 functions in murine brain and liver tissues. However, those assumptions have not yet been evaluated at the molecular level between TST and KEAP1 cysteines; therefore, additional in-depth investigations are required to fully understand this molecular interaction. Beyond this mechanism, activation of the TST pathway by, for instance, thiosulfate leads to activation of the NRF2 signaling pathway, demonstrating the potential of small molecule interactions to therapeutically target this pathway [42,114] (see Fig. 1).

**Table 1:** Abbreviations.

ACO1/2	Aconitase
AHS	Alpers–Huttenlocher syndrome
BMI	Body mass index
CBS	Cystathionine beta synthase
CSE	Cystathionine gamma lyase
CAT	Cysteine aminotransferase
CAP1A	Carnitine palmitoyltransferase 1a
CAVD	Calcific aortic valve disease
CKD	Chronic kidney disease
DHLA	Dihydrolipoic acid
ETC	Electron transport chain
ETHE1/PDO	Persulfide dioxygenase
FAO	Fatty acid oxidation
FRDA	Friedreich's ataxia
FADH2	Flavin adenine dinucleotide
GSH	Glutathione (reduced form)
GPX	Glutathione peroxidase
GSSG	Glutathione disulfide (oxidized glutathione)
GSSH	Glutathione persulfide
GR	Glutathione reductase
GCLC	Glutamate cysteine ligase, catalytic subunit
GCLM	Glutamate cysteine ligase, modifier subunit
IBD	Inflammatory bowel disease
H_2_S	Hydrogen sulfide
HMOX1	Heme Oxygenase 1
HDL	High-density lipoprotein
KEAP1	Kelch-like ECH-associated protein 1
KSS	Kearns–Sayre syndrome
LHON	Leber's Hereditary Optic Neuropathy
MD	Mitochondrial diseases
MDA	Malondialdehyde
MPO	Myelopeoxidase
MPST	3-mercaptopyruvate sulfurtransferase
NADH	Nicotinamide adenine dinucleotide (reduced form)
NADPH	Nicotinamide adenine dinucleotide phosphate (reduced form)
NRF2	Nuclear factor erythroid 2-related factor 2
OXPHOS	Oxidative phosphorylation
2-PTS	2-Propenyl thiosulfate
ROS	Reactive oxygen species
RSS	Reactive sulfur species
RSI	Reactive species interactome
RCS	Reactive carbon species
RNS	Reactive nitrogen species
SOD	Superoxide dismutase
SQOR	Sulfide quinone oxidoreductase
TCA cycle	Tricarboxylic acid cycle
TST	Thiosulfate sulfurtransferase
TXN	Thioredoxin
TXNRD	Thioredoxin reductase
VLCAD	Very long-chain specific acyl-CoA dehydrogenase

## 4 Changes of TST expression in preclinical cell and animal models

TST has been studied in preclinical models for various diseases, revealing its critical role in cellular defense against oxidative stress and metabolic dysfunction. The enzyme's function centers around sulfide metabolism, regulation of mitochondrial activity and protection against oxidative stress, which are implicated in a wide range of diseases. In Table 2, Table 3 we summarize its expression in various models including *in vivo* and *in vitro* studies.

**Table 2:** Changes of TST expression/activity in various pre-clinical disease models in cells.

Relevant function	Cell type	Expression/activity change	Disease model	Ref.
Sulfide metabolism	Valvular interstitial cells	↑	Calcific aortic valve disease	155
Sulfide metabolism	MCF-12A and MCF-7 cell line	↓	Breast cancer	156
Sulfide metabolism	6 human leukemia cell lines	↑	Leukemia	157
Sulfide metabolism; antioxidant	Normal astrocytes and U373 cell line	↑	Astrocytoma	158
Mitochondrial function regulation; antioxidant	Murine bone marrow-derived macrophages (BMDMs)	↑	Obesity	94
Mitochondrial function regulation	HK-2 cell line	↓	Diabetic kidney disease	117
Sulfide metabolism	Human colonic epithelial cell organoid	↑	Colon cancer	159

↑/↓: increase/decrease in protein/mRNA expression or enzyme activity when compared to the normal/healthy/younger condition.

**Table 3:** Changes of TST expression/activity in various pre-clinical disease models in animals.

Relevant function	Tissue/Organ	Expression/activity change	Disease model	Ref.
Sulfide metabolism	Rat kidney	↓	Hypertension and aging	160
Sulfide metabolism; antioxidant	Rat mesenteric adipose tissue	↓	High fructose diet-induced injury and aging	161
Mitochondrial function regulation	Mouse liver and plasma	↑	Dysregulation of high-density lipoprotein (HDL)	162
Antioxidant	Mice liver	↑	Low-dose radiation exposure	163
Sulfide metabolism; antioxidant	Rat stomach	↓	High fructose diet-induced gastric malfunction and aging	164
Sulfide metabolism	Rat liver	↑	Hypertension and aging	165
Sulfide metabolism; antioxidant	Rat liver	↓	Obesity	166

↑/↓: increase/decrease in protein/mRNA expression or enzyme activity when compared to the normal/healthy/younger condition.

Taken together, these studies position TST as a crucial mediator of various preclinical disease models, with beneficial effects across a variety of tissues and cell lines, ranging from adipose, liver, kidney, and stomach to brain. The ability of TST to modulate sulfide metabolism, interact with key metabolic pathways and exert anti-oxidative effects underscores its significance in both preclinical models and potential clinical applications for conditions related to those biological processes.

## 5 TST expression profiles in human diseases with mitochondrial dysfunction

Publicly available RNA sequencing and proteomic databases, including the GTEx project and the Human Protein Atlas (HPA) [115,116], reveal that TST is highly expressed in many organs with high metabolic activity such as liver, colon, kidney and brain. Recent studies have uncovered its diverse roles in disease pathology, ranging from metabolic disorders to obesity, neurological, cardiovascular, colonic diseases and cancers.

### 5.1 TST in metabolic health and obesity

Recently, a dramatic increase in obesity and in type 2 diabetes mellitus (T2DM) has been observed worldwide. TST has attracted significant attention for its role in adipose tissue biology and systemic metabolic regulation. In murine models, elevated TST expression in adipocytes has been shown to confer protection against obesity and T2DM [87]. In a cohort of almost 700 people, Morton et al. have extended these findings by reporting a negative correlation between TST activity with BMI, obesity and insulin resistance in adipose tissue, and a positive correlation with insulin receptor substrate 1 (IRS1) and adiponectin [87]. Adiponectin, a hormone with anti-inflammatory and insulin-sensitizing properties, is a critical mediator of metabolic homeostasis, and its positive association with TST suggests a protective role of this enzyme for human metabolic health [117,118]. In adipocytes, TST positively correlates with insulin receptor substrate 1 (IRS1) levels and enhances adiponectin secretion, a key adipokine that promotes insulin sensitivity [87]. Experimental evidence has shown that treatment with thiosulfate, a substrate for TST, increases adiponectin secretion from differentiated human adipocytes, further supporting its role in glucose homeostasis and lipid metabolism (Morton, 2016). Additionally, unchanged hepatic insulin sensitivity markers and impaired glucose tolerance were described in Tst^−/−^ mice [39]. Mechanistically, the maintenance of a healthy oxidation status in adipocytes is crucial to its normal functions [119], thiosulfate administration for TST activation suppressed the inflammatory response and upregulated Irs1 mRNA level in 3T3-L1 clonal adipocytes, supported by decreased Ccl2 and Il6 mRNA levels compared to palmitate-induced inflammation adipocytes [94]. Additionally, TST contributes to redox homeostasis by modulating reactive sulfur species (10.13039/100012100RSS) and ROS levels, which play a crucial role in metabolic health [43]. Along with those observations, endogenous and exogenous sulfide administration have been reported to influence hepatic glucose and lipid metabolism [120,121], in Tst^−/−^ mice.Increased hepatic gluconeogenesis was also detected in Tst^−/−^ mice compared with healthy mice [39]. Therefore, TST has been implicated in metabolic regulation through its effects on redox balance and sulfide metabolism. Thus, TST is emerging as a key metabolic regulator with potential applications as a biomarker for metabolic disorders, including obesity and diabetes.

### 5.2 TST in neurological-related disorders

TST deficiency has been implicated in mitochondrial dysfunction, notably in Leber's Hereditary Optic Neuropathy (LHON), a rare neurodegenerative disease characterized by severe vision loss [122]. LHON is primarily caused by mtDNA mutations that affect complex I of the ETC, resulting in increased production of ROS and consequent mitochondrial dysfunction [123]. TST functions in detoxifying cyanide and regulating mitochondrial ROS levels, and its deficiency exacerbates mitochondrial dysfunction in LHON patients. Evidence of TST deficiency in both liver and rectal tissues from LHON patients suggests that the enzyme plays a role in the systemic pathology of the disease [124]. Although conflicting data exist on the tissue-specificity of TST expression patterns in LHON patients, the enzyme's involvement in mitochondrial health remains significant [123]. Studies have shown reduced expression of both serine hydroxymethyltransferase and rhodanese in fibroblasts and lymphoblasts derived from FRDA patients [125].

### 5.3 TST in cardiovascular and kidney diseases

The functions of TST in mitigating oxidative stress and maintaining redox homeostasis are increasingly recognized as central to the pathogenesis of atherosclerosis, which further contributes to ischemic stroke and chronic kidney disease (CKD) [126,127]. Atherosclerosis is associated with vascular inflammation mediated by ROS and subsequent oxidative stress [128]. TST, along with TSTD2 (a TST-like enzyme), catalyzes sulfur transfer reactions and contributes to the reduction of antioxidants such as glutathione and thioredoxin, which are essential in detoxifying ROS and regulating cellular homeostasis. Elevated levels of TSTD2 autoantibodies have been identified in patients with atherosclerosis and CKD, suggesting that the body mounts an immune response against this sulfurtransferase because of oxidative stress and vascular damage. The presence of these autoantibodies correlates with known atherosclerosis risk factors, such as hypertension, smoking, and hyperglycemia. These findings imply that TST and its isoforms may be involved in the progression of vascular endothelial damage caused by ROS, positioning TST as a potential biomarker and therapeutic target for the prevention and management of atherosclerosis and CKD [129].

Furthermore, in diabetic kidney disease (DKD), TST deficiency contributes to the disruption of fatty acid oxidation (FAO), a critical process for energy production in renal tubular cells [117,126]. Decreased TST expression has been reported in renal tubular cells from patients with DKD. Specifically, TST downregulation leads to decreased S-sulfhydration of very long-chain specific acyl-CoA dehydrogenase (VLCAD), an enzyme essential for mitochondrial FAO. This reduction in S-sulfhydration impairs VLCAD activity, further exacerbating mitochondrial dysfunction and leading to defective fatty acid metabolism [130]. Conversely, interventions that restore TST activity, such as sodium thiosulfate treatment or TST overexpression, significantly alleviate renal tubular injury under high-glucose conditions [130]. These findings underscore the protective role of TST in preventing mitochondrial FAO dysfunction and its associated tubular damage [130].

Calcific aortic valve disease (CAVD) is the most prevalent form of valvular heart disease, characterized by systemic endothelial dysfunction [131] and significant rates of morbidity and mortality [132], whereby the dysregulation of sulfur metabolism and redox homeostasis exacerbate the calcification processes. The altered sulfide metabolism in this disease increased the interest to study H_2_S-related proteins including TST. TST was found to be upregulated in calcific human aortic valves, as a response to calcifying stimuli in CAVD [133]. This finding consolidated TST's role in sulfur trafficking within the mitochondria, which may further promote calcification in aortic valve tissues. Additionally, the accumulation of ROS in valve tissues, driven by a decline in TST function, may enhance inflammatory signaling and oxidative damage, which are known contributors to valvular calcification.

### 5.4 TST in inflammatory and gastrointestinal diseases

TST, has been shown to decrease in expression with age across multiple organs, including the colon [134]. In both pediatric and adult IBD patients, lower expression of H_2_S-metabolizing enzymes like TST has been consistently observed in both human and animal models [[135], [136], [137]]. This reduction may lead to impaired detoxification of H_2_S, resulting in elevated levels of the gas in the colon, which destabilizes the protective mucosal layer and promotes bacterial interactions with epithelial cells [136]. These interactions increase susceptibility to inflammation, worsening the disease. Furthermore, lower levels of TST could hinder the anti-inflammatory and protective effects of H_2_S, exacerbating intestinal damage and contributing to the chronic inflammation seen in IBD [137]. Interestingly, mucosal healing is associated with increased TST expression, suggesting that TST may play a reparative role in the gastrointestinal mucosa [135].

### 5.5 TST in cancer

TST plays a significant role in cancer biology due to its involvement in the sulfide metabolism pathway. H_2_S has been indicated as a regulator of tumor progression and metastasis in recent years [138], therefore, the disruption of sulfur metabolism through TST mutations can alter cellular redox states, leading to oxidative stress and changes in H_2_S levels. Emerging evidence suggests that TST dysregulation may contribute to cancer pathophysiology, particularly through its role in redox homeostasis and sulfur metabolism. In colorectal cancer, TSTD1 expression is significantly upregulated for sulfide homeostasis in patient tissues [51]. In addition, TSTD1 protein was highly expressed in 68.8 % of breast cancer patients from Taiwanese and Korean cohorts, and its overexpression in tumors was significantly correlated with reduced 5-year survival [139]. This dysregulation of sulfide metabolism may create an environment favorable for cancer progression, highlighting the enzyme as a potential target for cancer therapies [140,141].

**Fig. 2:**
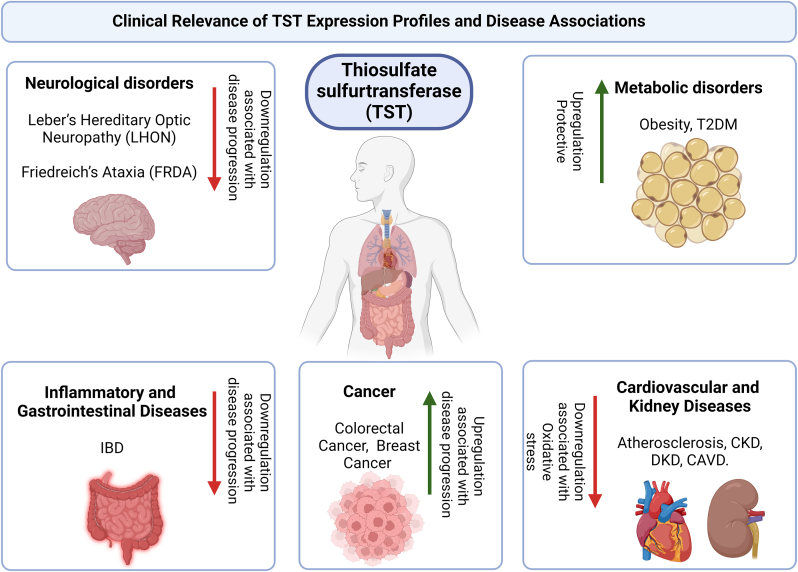
Clinical relevance of TST expression profiles and disease associations.

Fig. 2 illustrates the clinical relevance of thiosulfate sulfurtransferase (TST) expression profiles across various organ systems and its association with specific diseases. In adipose tissue, elevated TST expression is protective against obesity and type 2 diabetes mellitus (T2DM), showing positive correlations with metabolic health markers such as insulin receptor substrate 1 (IRS1) and adiponectin. In the brain, TST deficiency is linked to neurodegenerative conditions like Leber's Hereditary Optic Neuropathy (LHON) and Friedreich's ataxia (FRDA), where downregulated TST disrupts mitochondrial function and redox balance. Within the heart and kidneys, TST plays a protective role in cardiovascular diseases and chronic kidney disease (CKD) by supporting redox homeostasis, while in diabetic kidney disease (DKD), its deficiency impairs fatty acid oxidation, contributing to renal damage. In the colon, decreased TST expression is observed in inflammatory bowel disease (IBD), potentially reducing H_2_S detoxification, compromising the mucosal barrier, and increasing inflammation. In cancer, TST dysregulation affects redox balance and H_2_S metabolism, potentially promoting tumor progression and suggesting TST's role as a biomarker and therapeutic target in oncology.

## 6 Therapeutic options to activate the TST pathway

After elucidating the beneficial roles of TST in multiple diseases, activation of the TST pathway, thereby stimulating mitochondrial function and reducing oxidative stress, could be a novel and exciting therapeutic option for the prevention or attenuation of disease processes. This section summarizes how targeting TST might be useful for future clinical directions. For activators of TST, the experimental options currently available in the literature are: sodium thiosulfate, a novel small molecule (Hit 2), and hTST protein [25,[142], [143], [144]].

### 6.1 Potential clinical and present clinical applications of sodium thiosulfate

Sodium thiosulfate (hereafter referred to as STS or thiosulfate) is an endogenous sulfur metabolite [145], which possesses antioxidant [146], anti-inflammatory [94], and antihypertensive properties [147]. It has a long history of medicinal use in metal and cyanide intoxications and, more recently, in the prevention of ototoxicity of cisplatin. STS also has the potential for TST activation and intracellular H_2_S generation [148,149], suggesting therapeutic potential beyond its current clinical use (see Table 5). The activation of TST expression by thiosulfate has been validated in many studies [94,150,151], and the beneficial effects of STS for intracellular H_2_S generation have been summarized elsewhere [143]. Sodium thiosulfate is a water-soluble, inorganic compound with no detectable odor, characterized by the chemical formula Na_2_S_2_O_3_ and a molecular weight of 158.11 g/mol. S_2_O_3_^2−^ can be generated endogenously by several other mechanisms including the oxidation of H_2_S, and the interaction between sulfite and sulfane Sulfur [152,153]. Thiosulfate has been traditionally used clinically as an antidote for cyanide poisoning with the reaction mentioned in **Section 2**. In recent years, STS was proposed for further therapeutic uses [154], some of which are summarized in Table 4, Table 5, Table 6.

**Table 4:** Cytotoxic and cytoprotective effects of STS in cultured cells.

Cell type	Disease model	Concentration	Cellular response elicited by STS	Ref.
The human keratinocyte (HaCaT) cell line	Atopic dermatitis	2.0 mmol/L	Decreased IL-1β and IL-6 mRNA expression; increased MnSOD activity	167
Isolated PBMCs	Hemorrhagic shock in a porcine model	25 mg/kg/h for 2 h	Increased mitochondrial oxygen consumption; increased acetyl-CoA flux	168
B16 and A375 cells	Skin cancer	STS (0, 0.25, 1,2.5 mM) for 24 h or 48 h	Reduced cell proliferation, viability, and EMT process; increased H_2_S, down-regulated Wnt/β-catenin pathway	169
Pooled human umbilical vein endothelial cells (HUVECs)	Vascular occlusive diseases	4 h with 3 mM STS	Decreased mitochondrial respiration; increased glycolysis and ATP production	144
HUVEC and human lung microvascular endothelial cells (HMVEC-L)	Acute lung injury	5–20 mM for 20h	Deceased ROS production; inhibited IKK/NFκB activation	146
Primary human vascular smooth muscle cells (VSMCs)	Intimal hyperplasia	3 or 15 mM STS for 24h∼7days	Impaired proliferation, migration and ECM-secreting phenotype; inhibited microtube polymerization	170
Rat renal epithelial (NRK-52E) cell	Hypoxia–reoxygenation injury	150 μM STS for 2 h	STS pre-treatment alone attenuated cell apoptosis	171
Human neuroblastoma (SH-SY5Y) cell line; primary cortical neurons from C57BL/6J mice	Neuronal ischemia reperfusion injury	0.25 mmol/L for 24h	Increased thiosulfate level and GSH contents; inhibited apoptotic pathway via modulating JNK pathway and Erk1/2 pathway	55
Preadipocyte cell line model (3T3-L1)	Obesity	50 mM STS	Reduced palmitate-induced inflammation; improved insulin tolerance and anti-oxidative damage function	94

**Table 5:** Effects of STS in animal models.

Animal	Indicated disease	Concentration	Response elicited by STS	Ref.
BALB/C female mice	Atopic dermartitis	1.28 mg/mL for 7 days	Decreased the infiltration of dermal inflammatory cells; decreased ROS and inflammatory cytokines expression	172
C57BL/6J and Cystathionine γ-lyase (CSE) knockout mice	Hepatic ischemic and reperfusion (I/R) injury	0.015 mmol/L, 0.15 mmol/L, and 1.5 mmol/L, reperfusion of 1 or 24 h	Increased GSH amount and mitochondrial integrity	173
C57BL/6JRj mice and LDLR^−/−^ mice	Vascular occlusive diseases	0.5 or 1 g/kg/day, 3 injections per week	Increased reperfusion and muscle recovery; increased H_2_S production and protein persulfidation	144
C57BL/6J mice	Acute lung injury	Intraperitoneal administration of 2 g/kg STS at 0 and 12h after intratracheal LPS	Attenuated the pulmonary vascular leakage and lung edema; decreased IL-6, IL-1β and TNFα	146
Wistar rats	DOX-induced cardiotoxicity during cancer treatment	300 mg/kg, 3 times per week, i.p	Improved the body weight and cardiotoxicity; decreased oxidative stress: increased GSH, SOD and decreased lipid peroxidation	174
WT, LDLR^−/−^ mice, and Cse^−/−^ mice	Intimal hyperplasia (IH)	4 g/L (0.5 g/Kg/day), changed 3 times a week	Reduced IH in WT and LDLR^−/−^ mice; STS fully rescued CSE^−/−^ mice from increased IH	170
Sprague–Dawley (SD) rats	Hypertension with adenine-induced chronic kidney disease	2 g/kg body weight/day for 2 weeks	Reduced blood pressure; increased levels of H_2_S and thiosulfate in plasma; increased NO bioavailability	175
C57BL/6J mice	Neuronal ischemia reperfusion injury	10 mg/kg (40 umol/kg)	Improved the 20-day survival rate of mice subjected to bilateral common carotid artery occlusion	55
Pdx1 knockdown zebrafish	Hyperglycemia induced kidney damage	10 mM	Restored glomerular enlargement; reduced pronephric neck length in *pdx1* morphants	25
Lewis rats	Syngeneic kidney transplantation	Donor rats were pre-treated with 2.4 mg STS/kg for 30mins; the procured renal grafts were stored for 24h	Reduced apoptosis levels in renal grafts after transplantation	171

**Table 6:** Clinically used and approved STS in human diseases.

Sodium thiosulfate	Targeting mechanism	Disease	Effective range	Development stage	Ref.
	TST substrate, sulfur donor	Acute cyanide poisoning	250 mg/mL in an “antidote kit”	Clinical use since the 1930s	[154,176]
	Antioxidant; reaction with cisplatin structure	Reducing cisplatin-induced hearing loss in pediatric cancer patients	At a dose of 20 g per square meter	FDA-approved	[177,178]
	Calcium-chelating agent, binding to Ca^2+^ and increasing its solubility.	Calciphylaxis in dialysis patients	20 g of STS infusion for 4 days; 25 g of STS was also administered intravenously	Off-label drug	[16,[179], [180], [181]]

STS can be safely administered to humans by i.v. administration and is already a clinically viable molecule approved by the FDA [182]. Although STS can be administered with the drinking water, the acid environment of the stomach will degrade a significant part of the compound. To ensure long-term treatment an oral formulation has recently been developed and patented (European Patent Application No. 23701222.4; U.S. patent application No. 18/729,995) which will soon be tested in human disease conditions. The new formula ensures slow release and is acid resistant. As an additional feature, it will maintain its slow-release potential if it breaks down one way or another. We believe that by activating the TST pathway a protective mechanism will be set in motion in diseases associated with oxidative stress, mitochondrial dysfunction and aberrant redox signaling.

### 6.2 Preclinical studies for specific TST activation

**Fig. 3:**
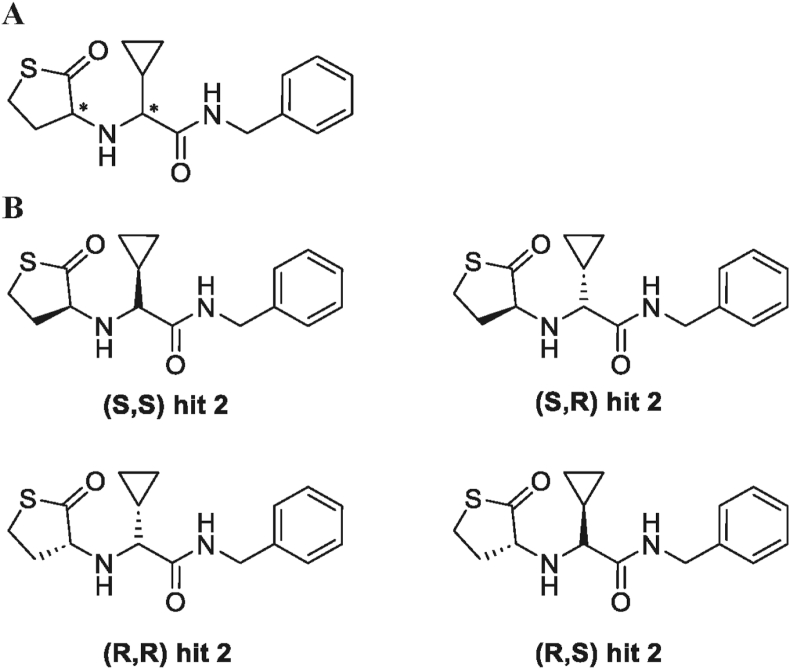
A: Chemical structure of Hit 2, which contains two stereo-centers (indicated by 2 asterisks). **B**: While the identity of the first stereo center (left∗) can be thereby be determined, assignment of the second stereocenter (right∗) cannot be made from the data from the chiral column alone.

In order to have a more specific and potent activation of TST, there are two developing strategies as hTST protein and a small molecule (Hit 2: TST activator) [142]. The hTST protein was used as a reagent for TST activation in Pdx1 knockdown zebrafish to reduce oxidative stress. A concentration of 1 μg/μL of protein was applied, which resulted in the restoration of glomerular enlargement and a reduction in pronephric neck length in pdx1 morphants [25]. Additionally, Hit 2 was used as a reagent for TST activation in C57BL/6J mice, specifically in isolated mitochondria from brain tissue. A concentration of 50 μM of Hit 2 was administered, leading to an increase in maximum uncoupled respiration and state 3 respiration [142]. The chemical structure of Hit 2 is shown in Fig. 3.

The calculation of the binding free energy for Hit 2 indicates that the van der Waals component is the predominant contributor to the total binding energy [142]. Per-residue energy decomposition analysis reveals that the binding of Hit 2 to hTST is primarily stabilized by energetically favorable interactions with residues Leu6, Ala9, Leu10, and Tyr262. The potential activation of hTST by Hit 2 may be attributed to an enhanced substrate-binding environment within the catalytic site, characterized by increased accessibility of the binding pocket and improved stabilization of substrate-derived negative charges by positively charged residues in the catalytic site. The detailed interaction are mentioned in a previous study [142]. However, the protein structure was modeled using the primary sequence from UniProt (Q16762), there is no accurate structure of hTST protein yet, this compound needs further validation and investigation on human TST structure for further studies.

## 7 Conclusion and directions for drug exploration

The current review highlights TST's diverse functionality and clinical relevance. Pharmacological research related to TST is a rapidly advancing field, with the potential to generate numerous therapeutic opportunities, and besides STS, early-stage drug candidates are now under investigation (Section 6.2). Additionally, overexpression of TST as a therapeutic modality might be explored. A transgenic mouse with selective adipose overexpression of TST has recently been used to confirm the anti-diabetic phenotype with improved hepatic fat oxidation [87]. Therefore, despite its known protective roles within mitochondria and possible interaction with NRF2 signaling, the overexpression and activation of TST have received limited attention as a potential therapeutic strategy to address diseases associated with mitochondrial dysfunction.

## CRediT authorship contribution statement

**Yang Luo:** Writing – review & editing, Writing – original draft, Visualization, Project administration, Conceptualization. **Shaden Melhem:** Writing – review & editing, Writing – original draft, Visualization, Investigation, Conceptualization. **Martin Feelisch:** Writing – review & editing. **Laurent Chatre:** Writing – review & editing. **Nicholas M. Morton:** Writing – review & editing, Supervision. **Amalia M. Dolga:** Writing – review & editing, Supervision. **Harry van Goor:** Writing – review & editing, Supervision, Resources, Project administration, Conceptualization.

## Funding

Y.L. was supported by China Scholarship Council (Grant No.: 202008520033). A.M.D. was supported by a Rosalind Franklin Fellowship co-funded by the European Union and the University of Groningen. S.M. was supported by a British Heart Foundation 4Y PhD Scholarship (FS/4yPhD/F/20/34,126). L.C was supported by the CNRS.

## Declaration of competing interest

The authors declare that they have no known competing financial interests or personal relationships that could have appeared to influence the work reported in this paper.

## Data Availability

No data was used for the research described in the article.

## References

[1] Duchen M.R. (2004). Mitochondria in health and disease: perspectives on a new mitochondrial biology. Mol. Aspect. Med..

[2] Nunnari J., Suomalainen A. (2012). Mitochondria: in sickness and in health. Cell.

[3] Dolezal P., Likic V., Tachezy J., Lithgow T. (1979). Evolution of the molecular machines for protein import into mitochondria. Science.

[4] Hughes D.A., Jastroch M., Stoneking M., Klingenspor M. (2009). Molecular evolution of UCP1 and the evolutionary history of mammalian non-shivering thermogenesis. BMC Evol. Biol..

[5] Hopper R.K., Carroll S., Aponte A.M. (2006). Mitochondrial matrix phosphoproteome: effect of extra mitochondrial calcium. Biochemistry.

[6] Antonucci S., Di Lisa F., Kaludercic N. (2021). Mitochondrial reactive oxygen species in physiology and disease. Cell Calcium.

[7] Peoples J.N., Saraf A., Ghazal N., Pham T.T., Kwong J.Q. (2019). Mitochondrial dysfunction and oxidative stress in heart disease. Exp. Mol. Med..

[8] Gorman G.S., Chinnery P.F., DiMauro S. (2016). Mitochondrial diseases. Nat. Rev. Dis. Primers.

[9] McFarland R., Taylor R.W., Turnbull D.M. (2010). A neurological perspective on mitochondrial disease. Lancet Neurol..

[10] Scarpelli M., Todeschini A., Volonghi I., Padovani A., Filosto M. (2017). Mitochondrial diseases: advances and issues. Appl. Clin. Genet..

[11] El-Hattab A.W., Emrick L.T., Craigen W.J., Scaglia F. (2012). Citrulline and arginine utility in treating nitric oxide deficiency in mitochondrial disorders. Mol. Genet. Metabol..

[12] Buonvino S., Arciero I., Melino S. (2022). Thiosulfate-cyanide sulfurtransferase a mitochondrial essential enzyme: from cell metabolism to the biotechnological applications. Int. J. Mol. Sci..

[13] Bordo D., Bork P. (2002). The rhodanese/Cdc25 phosphatase superfamily. EMBO Rep..

[14] Alsohaibani R., Claudel A.L., Perchat-Varlet R. (2023). Rhodanese-Fold containing proteins in humans: not just key players in sulfur trafficking. Antioxidants.

[15] Cicero D.O., Melino S., Orsale M. (2003). Structural rearrangements of the two domains of Azotobacter vinelandii rhodanese upon sulfane sulfur release: essential molecular dynamics, NMR relaxation and deuterium exchange on the uniformly labeled protein. Int. J. Biol. Macromol..

[16] Kruithof P.D., Lunev S., Aguilar Lozano S.P. (2020). Unraveling the role of thiosulfate sulfurtransferase in metabolic diseases. Biochim. Biophys. Acta, Mol. Basis Dis..

[17] Spinelli J.B., Haigis M.C. (2018). The multifaceted contributions of mitochondria to cellular metabolism. Nat. Cell Biol..

[18] Nolfi-Donegan D., Braganza A., Shiva S. (2020). Mitochondrial electron transport chain: oxidative phosphorylation, oxidant production, and methods of measurement. Redox Biol..

[19] Lang K. (1933). Die rhodanbildung im tierkorper [thiocyanogen in the bodies of animals]. Biochem. Z..

[20] Kaleta K., Misterka A., Rydz L., Wróbel M., Jurkowska H. (2023). Correlation between the level of sulfane sulfur and the expression/activity of sulfurtransferases in chicken tissues – a possible ways of cyanide detoxification. Biologia (Bratisl)..

[21] Zuhra K., Szabo C. (2022). The two faces of cyanide: an environmental toxin and a potential novel mammalian gasotransmitter. FEBS J..

[22] Whitehouse D.B., Pilz A.J., Porta G., Hopkinson D.A. (1988). Rhodanese isozymes in human tissues. Ann. Hum. Genet..

[23] Augsburger F., Szabo C. (2020). Potential role of the 3-mercaptopyruvate sulfurtransferase (3-MST)—hydrogen sulfide (H2S) pathway in cancer cells. Pharmacol. Res..

[24] Williams R.A.M., Kelly S.M., Mottram J.C., Coombs G.H. (2003). 3-Mercaptopyruvate sulfurtransferase of LeishmaniaContains an unusual C-terminal extension and is involved in thioredoxin and antioxidant metabolism. J. Biol. Chem..

[25] Al-Dahmani Z.M., Li X., Wiggenhauser L.M. (2022). Thiosulfate sulfurtransferase prevents hyperglycemic damage to the zebrafish pronephros in an experimental model for diabetes. Sci. Rep..

[26] Rydz L., Wróbel M., Jurkowska H. (2021). Sulfur administration in Fe–S cluster homeostasis. Antioxidants.

[27] Sousa F.M., Pereira J.G., Marreiros B.C., Pereira M.M. (2018). Taxonomic distribution, structure/function relationship and metabolic context of the two families of sulfide dehydrogenases: SQR and FCSD. Biochim. Biophys. Acta Bioenerg..

[28] Zhou Z., Tran P.Q., Cowley E.S., Trembath-Reichert E., Anantharaman K. (2024). Diversity and ecology of microbial sulfur metabolism. Nat. Rev. Microbiol..

[29] Rydz L., Wróbel M., Jurkowska H. (2021). Sulfur administration in Fe–S cluster homeostasis. Antioxidants.

[30] Brosnan J.T., Brosnan M.E. (2006). The sulfur-containing amino acids: an overview. J. Nutr..

[31] Kimura H. (2015). Signaling molecules: hydrogen sulfide and polysulfide. Antioxidants Redox Signal..

[32] Abe K., Kimura H. (1996). The possible role of hydrogen sulfide as an endogenous neuromodulator. J. Neurosci..

[33] Goodwin L.R., Francom D., Dieken F.P. (1989). Determination of sulfide in brain tissue by gas dialysis/ion chromatography: postmortem studies and two case reports. J. Anal. Toxicol..

[34] Blackstone E., Morrison M., Roth M.B. (2005). H2S induces a suspended animation-like state in mice. Science.

[35] Renga B. (2011). Hydrogen sulfide generation in mammals: the molecular biology of cystathionine-β-synthase (CBS) and cystathionine-γ-lyase (CSE). Inflamm. Allergy - Drug Targets.

[36] Zainol Abidin QH., Ida T., Morita M. (2023). Synthesis of sulfides and persulfides is not impeded by disruption of three canonical enzymes in sulfur metabolism. Antioxidants.

[37] Shibuya N., Tanaka M., Yoshida M. (2009). 3-Mercaptopyruvate sulfurtransferase produces hydrogen sulfide and bound sulfane sulfur in the brain. Antioxidants Redox Signal..

[38] Hildebrandt T.M., Grieshaber M.K. (2008). Three enzymatic activities catalyze the oxidation of sulfide to thiosulfate in mammalian and invertebrate mitochondria. FEBS J..

[39] Carter R.N., Gibbins M.T.G., Barrios-Llerena M.E. (2021). The hepatic compensatory response to elevated systemic sulfide promotes diabetes. Cell Rep..

[40] Vitvitsky V., Yadav P.K., An S., Seravalli J., Cho U.S., Banerjee R. (2017). Structural and mechanistic insights into hemoglobin-catalyzed hydrogen sulfide oxidation and the fate of polysulfide products. J. Biol. Chem..

[41] Vitvitsky V., Yadav P.K., Kurthen A., Banerjee R. (2015). Sulfide oxidation by a noncanonical pathway in red blood cells generates thiosulfate and polysulfides. J. Biol. Chem..

[42] Zhang M.Y., Dugbartey G.J., Juriasingani S., Sener A. (2021). Hydrogen sulfide metabolite, sodium thiosulfate: clinical applications and underlying molecular mechanisms. Int. J. Mol. Sci..

[43] Luo Y., Chatre L., Melhem S. (2023). Thiosulfate sulfurtransferase deficiency promotes oxidative distress and aberrant NRF2 function in the brain. Redox Biol..

[44] Villarejol M., Westley J. (1963). Rhodanese-catalyzed reduction of thiosulfate by reduced lipoic acid. J. Biol. Chem..

[45] Iciek M., Kowalczyk-Pachel D., Bilska-Wilkosz A., Kwiecién I., Górny M., Wøodek L. (2016). S-sulfhydration as a cellular redox regulation. Biosci. Rep..

[46] Libiad M., Sriraman A., Banerjee R. (2015). Polymorphic variants of human rhodanese exhibit differences in thermal stability and sulfur transfer kinetics. J. Biol. Chem..

[47] Sies H. (2020). Findings in redox biology: from H2O2 to oxidative stress. J. Biol. Chem..

[48] Jones D.P. (2006). Redefining oxidative stress. Antioxidants Redox Signal..

[49] Xie Z.Z., Liu Y., Bian J.S. (2016). Hydrogen sulfide and cellular redox homeostasis. Oxid. Med. Cell. Longev..

[50] Chatgilialoglu C., Bowry V.W. (2018). Why not trans? Inhibited radical isomerization cycles and coupling chains of lipids and alkenes with alkane *-* thiols. J. Org. Chem..

[51] Libiad M., Motl N., Akey D.L. (2018). Thiosulfate sulfurtransferase-like domain-containing 1 protein interacts with thioredoxin. J. Biol. Chem..

[52] Benchoam D., Cuevasanta E., Roman J.V., Banerjee R., Alvarez B. (2024). Acidity of persulfides and its modulation by the protein environments in sulfide quinone oxidoreductase and thiosulfate sulfurtransferase. J. Biol. Chem..

[53] Libiad M., Yadav P.K., Vitvitsky V., Martinov M., Banerjee R. (2014). Organization of the human mitochondrial hydrogen sulfide oxidation pathway. J. Biol. Chem..

[54] Lutchmansingh F.K., Hsu J.W., Bennett F.I. (2018). Glutathione metabolism in type 2 diabetes and its relationship with microvascular complications and glycemia. PLoS One.

[55] Marutani E., Yamada M., Ida T. (2015). Thiosulfate mediates cytoprotective effects of hydrogen sulfide against neuronal ischemia. J. Am. Heart Assoc..

[56] Lee S., Kim S.M., Lee R.T. (2013). Thioredoxin and thioredoxin target proteins: from molecular mechanisms to functional significance. Antioxidants Redox Signal..

[57] Tang H., Kim M., Lee M. (2022). Overexpression of thioredoxin‐2 attenuates age‐related muscle loss by suppressing mitochondrial oxidative stress and apoptosis. JCSM Rapid Commun.

[58] de Paula C.P., dos Santos M.C., Tairum C.A. (2020). Glutaredoxin-like protein (GLP)—a novel bacteria sulfurtransferase that protects cells against cyanide and oxidative stresses. Appl. Microbiol. Biotechnol..

[59] Higgins K.A., Peng H., Luebke J.L., Chang F.M.J., Giedroc D.P. (2015). Conformational analysis and chemical reactivity of the multidomain sulfurtransferase, *Staphylococcus aureus* CstA. Biochemistry.

[60] Nakajima T. (2015). Roles of sulfur metabolism and rhodanese in detoxification and anti-oxidative stress functions in the liver: responses to radiation exposure. Med. Sci. Monit..

[61] Henne M., König N., Triulzi T. (2015). Sulfurtransferase and thioredoxin specifically interact as demonstrated by bimolecular fluorescence complementation analysis and biochemical tests. FEBS Open Bio.

[62] Nandi D.L., Horowitz P.M., Westley J. (2000). Rhodanese as a thioredoxin oxidase. Int. J. Biochem. Cell Biol..

[63] Sabelli R., Iorio E., De Martino A. (2008). Rhodanese–thioredoxin system and allyl sulfur compounds. FEBS J..

[64] Libiad M., Motl N., Akey D.L. (2018). Thiosulfate sulfurtransferase-like domain–containing 1 protein interacts with thioredoxin. J. Biol. Chem..

[65] Nandi D.L., Horowitz P.M., Westley J. (2000). Rhodanese as a thioredoxin oxidase. Int. J. Biochem. Cell Biol..

[66] Luo Y., Chatre L., Al-Dahmani Z.M. (2023). Thiosulfate sulfurtransferase deficiency promotes oxidative distress in cerebral prefrontal cortex. Free Radic. Biol. Med..

[67] Mao Z., Huang Y., Zhang Z. (2019). Pharmacological levels of hydrogen sulfide inhibit oxidative cell injury through regulating the redox state of thioredoxin. Free Radic. Biol. Med..

[68] Wedmann R., Onderka C., Wei S. (2016). Improved tag-switch method reveals that thioredoxin acts as depersulfidase and controls the intracellular levels of protein persulfidation. Chem. Sci..

[69] Dóka É., Pader I., Bíró A. (2016). A novel persulfide detection method reveals protein persulfide- and polysulfide-reducing functions of thioredoxin and glutathione systems. Sci. Adv..

[70] Read A.D., Bentley R.E.T., Archer S.L., Dunham-Snary K.J. (2021). Mitochondrial iron–sulfur clusters: structure, function, and an emerging role in vascular biology. Redox Biol..

[71] Lanz N.D., Booker S.J. (2015). Auxiliary iron–sulfur cofactors in radical SAM enzymes. Biochim. Biophys. Acta Mol. Cell Res..

[72] Robbins A.H., Stout C.D. (1989). The structure of aconitase. Proteins: Struct., Funct., Bioinf..

[73] Hentze M.W., Kühn L.C. (1996). Molecular control of vertebrate iron metabolism: mRNA-based regulatory circuits operated by iron, nitric oxide, and oxidative stress. Proc. Natl. Acad. Sci. USA.

[74] Kiley P. (1998). Oxygen sensing by the global regulator, FNR: the role of the iron-sulfur cluster. FEMS Microbiol. Rev..

[75] Kobayashi K., Fujikawa M., Kozawa T. (2014). Oxidative stress sensing by the iron–sulfur cluster in the transcription factor, SoxR. J. Inorg. Biochem..

[76] Imlay J.A. (2006). Iron‐sulphur clusters and the problem with oxygen. Mol. Microbiol..

[77] Py B., Barras F. (2010). Building Fe–S proteins: bacterial strategies. Nat. Rev. Microbiol..

[78] Lu Z., Imlay J.A. (2019). A conserved motif liganding the [4Fe–4S] cluster in [4Fe–4S] fumarases prevents irreversible inactivation of the enzyme during hydrogen peroxide stress. Redox Biol..

[79] Bonomi F., Pagani S., Cerletti P., Cannella C. (1977). Rhodanese‐mediated sulfur transfer to succinate dehydrogenase. Eur. J. Biochem..

[80] Rydz L., Wróbel M., Jurkowska H. (2021). Sulfur administration in Fe–S cluster homeostasis. Antioxidants.

[81] Pagani S., Galante Y.M. (1983). Interaction of rhodanese with mitochondrial NADH dehydrogenase. Biochim. Biophys. Acta Protein Struct. Mol. Enzymol..

[82] Tomati U., Giovannozzi-Sermanni G., Duprè S., Cannella C. (1976). NADH: nitrate reductase activity restoration by rhodanese. Phytochemistry.

[83] Pagani S., Bonomi F., Cerletti P. (1982). Sulfide insertion into spinach ferredoxin by rhodanese. Biochim. Biophys. Acta Protein Struct. Mol. Enzymol..

[84] Pagani S., Bonomi F., Cerletti P. (1984). Enzymic synthesis of the iron‐sulfur cluster of spinach ferredoxin. Eur. J. Biochem..

[85] Pagani S., Eldridge M., Eady R.R. (1987). Nitrogenase of *Klebsiella pneumoniae* . Rhodanese-catalysed restoration of activity of the inactive 2Fe species of the Fe protein. Biochem. J..

[86] Taniguchi T., Kimura T. (1974). Role of 3-mercaptopyruvate sulfurtransferase in the formation of the iron-sulfur chromophore of adrenal ferredoxin. Biochim. Biophys. Acta Enzymol..

[87] Morton N.M., Beltram J., Carter R.N. (2016). Genetic identification of thiosulfate sulfurtransferase as an adipocyte-expressed antidiabetic target in mice selected for leanness. Nat. Med..

[88] Stamati K., Mudera V., Cheema U. (2011). Evolution of oxygen utilization in multicellular organisms and implications for cell signalling in tissue engineering. J. Tissue Eng..

[89] Lopez-Pascual A., Trayhurn P., Martínez J.A., González-Muniesa P. (2021). Oxygen in metabolic dysfunction and its therapeutic relevance. Antioxidants Redox Signal..

[90] Auten R.L., Davis J.M. (2009). Oxygen toxicity and reactive oxygen species: the devil is in the details. Pediatr. Res..

[91] Patti M.E., Corvera S. (2010). The role of mitochondria in the pathogenesis of type 2 diabetes. Endocr. Rev..

[92] Bourgonje A.R., Feelisch M., Faber K.N., Pasch A., Dijkstra G., van Goor H. (2020). Oxidative stress and redox-modulating therapeutics in Inflammatory bowel disease. Trends Mol. Med..

[93] Sies H., Jones D.P. (2020). Reactive oxygen species (ROS) as pleiotropic physiological signalling agents. Nat. Rev. Mol. Cell Biol..

[94] Lainšček D., Šuštar U., Carter R.N., Morton N.M., Horvat S. (2020). Tst gene mediates protection against palmitate-induced inflammation in 3T3-L1 adipocytes. Biochem. Biophys. Res. Commun..

[95] Iciek M., Górny M., Kotańska M., Bilska-Wilkosz A., Kaczor-Kamińska M., Zagajewski J. (2023). Yohimbine alleviates oxidative stress and suppresses aerobic cysteine metabolism elevated in the rat liver of high-fat diet-fed rats. Molecules.

[96] Zheng A., Li H., Feng Z., Liu J. (2021). Integrative analyses reveal Tstd1 as a potential modulator of HDL cholesterol and mitochondrial function in mice. Cells.

[97] Kang D., Lee J., Wu C. (2020). The role of selenium metabolism and selenoproteins in cartilage homeostasis and arthropathies. Exp. Mol. Med..

[98] Dunning B.J., Bourgonje A.R., Bulthuis M.L.C. (2023). Selenium and coenzyme Q10 improve the systemic redox status while reducing cardiovascular mortality in elderly population-based individuals. Free Radic. Biol. Med..

[99] Ogasawara Y., Lacourciere G., Stadtman T.C. (2001). Formation of a selenium-substituted rhodanese by reaction with selenite and glutathione: possible role of a protein perselenide in a selenium delivery system. Proc. Natl. Acad. Sci. USA.

[100] Cipollone R., Ascenzi P., Visca P. (2007). Common themes and variations in the rhodanese superfamily. IUBMB Life.

[101] Lee N., Park S.J., Lange M. (2024). Selenium reduction of ubiquinone via SQOR suppresses ferroptosis. Nat. Metab..

[102] Malard E., Valable S., Bernaudin M., Pérès E., Chatre L. (2021). The reactive species interactome in the brain. Antioxidants Redox Signal..

[103] Cortese-Krott M.M., Koning A., Kuhnle G.G.C. (2017). The reactive species interactome: evolutionary emergence, biological significance, and opportunities for redox metabolomics and personalized medicine. Antioxidants Redox Signal..

[104] Chatre L. (2024). Mitochondria and the reactive species interactome: shaping the future of mitoredox medicine. Journal of Mitochondria, Plastids and Endosymbiosis.

[105] Suzuki T., Yamamoto M. (2015). Molecular basis of the keap1–nrf2 system. Free Radic. Biol. Med..

[106] Saito R., Suzuki T., Hiramoto K. (2016). Characterizations of three major cysteine sensors of Keap1 in stress response. Mol. Cell Biol..

[107] Raghunath A., Sundarraj K., Nagarajan R. (2018). Antioxidant response elements: discovery, classes, regulation and potential applications. Redox Biol..

[108] Xie L., Gu Y., Wen M. (2016). Hydrogen sulfide induces Keap1 S-sulfhydration and suppresses diabetes-accelerated atherosclerosis via Nrf2 activation. Diabetes.

[109] Cuadrado A., Manda G., Hassan A. (2018). Transcription factor NRF2 as a therapeutic target for chronic diseases: a systems medicine approach. Pharmacol. Rev..

[110] Abdul-Aziz A., Macewan D.J., Bowles K.M., Rushworth S.A. (2015). Oxidative stress responses and NRF2 in human leukaemia. Oxid. Med. Cell. Longev..

[111] Yang G., Zhao K., Ju Y. (2013). Hydrogen sulfide protects against cellular senescence via s-sulfhydration of keap1 and activation of Nrf2. Antioxidants Redox Signal..

[112] Zhao S., Song T., Gu Y. (2021). Hydrogen sulfide alleviates liver injury through the S-Sulfhydrated-Kelch-Like ECH-associated protein 1/nuclear erythroid 2–related factor 2/low-density lipoprotein receptor–related protein 1 pathway. Hepatology.

[113] Hourihan J.M., Kenna J.G., Hayes J.D. (2013). The gasotransmitter hydrogen sulfide induces nrf2-target genes by inactivating the Keap1 ubiquitin ligase substrate adaptor through formation of a disulfide bond between cys-226 and cys-613. Antioxidants Redox Signal..

[114] Calvert J.W., Jha S., Gundewar S. (2009). Hydrogen sulfide mediates cardioprotection through Nrf2 signaling. Circ. Res..

[115] Lonsdale J., Thomas J., Salvatore M. (2013). The genotype-tissue expression (GTEx) project. Nat. Genet..

[116] Sjöstedt E., Zhong W., Fagerberg L. (2020). An atlas of the protein-coding genes in the human, pig, and mouse brain. Science.

[117] Zhang J.X., Chen P.P., Li X.Q. (2024). Deficiency of thiosulfate sulfurtransferase mediates the dysfunction of renal tubular mitochondrial fatty acid oxidation in diabetic kidney disease. Cell Death Differ..

[118] Khoramipour K., Chamari K., Hekmatikar A.A. (2021). Adiponectin: structure, physiological functions, role in diseases, and effects of nutrition. Nutrients.

[119] Kusminski C.M., Holland W.L., Sun K. (2012). MitoNEET-driven alterations in adipocyte mitochondrial activity reveal a crucial adaptive process that preserves insulin sensitivity in obesity. Nat. Med..

[120] Pichette J., Gagnon J. (2016). Implications of hydrogen sulfide in glucose regulation: how H2S can alter glucose homeostasis through metabolic hormones. Oxid. Med. Cell. Longev..

[121] Mani S., Cao W., Wu L., Wang R. (2014). Hydrogen sulfide and the liver. Nitric Oxide.

[122] Poole C.J., Kind P.R. (1986). Deficiency of thiosulphate sulphurtransferase (rhodanese) in Leber's hereditary optic neuropathy. Br. Med. J..

[123] Wallace D.C., Singh G., Lott M.T. (1988). Mitochondrial DNA mutation associated with Leber's hereditary optic neuropathy. Science.

[124] Cagianut B., Rhyner K., Furrer W., Schnebli H.P. (1981). Thiosulphate-sulphurtransferase (rhodanese) deficiency in Leber's hereditary optic atroph. Lancet.

[125] Tan G. (2003). Decreased expression of genes involved in sulfur amino acid metabolism in frataxin-deficient cells. Hum. Mol. Genet..

[126] Drüeke T.B., Massy Z.A. (2010). Atherosclerosis in CKD: differences from the general population. Nat. Rev. Nephrol..

[127] Baldassarre D., Castelnuovo S., Frigerio B. (2009). Effects of timing and extent of smoking, type of cigarettes, and concomitant risk factors on the association between smoking and subclinical atherosclerosis. Stroke.

[128] Batty M., Bennett M.R., Yu E. (2022). The role of oxidative stress in atherosclerosis. Cells.

[129] Kubota M., Zhang B.S., Li S.Y. (2022). Serum anti-TSTD2 antibody as a biomarker for atherosclerosis-induced ischemic stroke and chronic kidney disease. Med. Int..

[130] Zhang J.X., Chen P.P., Li X.Q. (2024). Deficiency of thiosulfate sulfurtransferase mediates the dysfunction of renal tubular mitochondrial fatty acid oxidation in diabetic kidney disease. Cell Death Differ..

[131] Quast C., Bönner F., Polzin A. (2024). Aortic valve stenosis causes accumulation of extracellular hemoglobin and systemic endothelial dysfunction. Circulation.

[132] Yutzey K.E., Demer L.L., Body S.C. (2014). Calcific aortic valve disease. Arterioscler. Thromb. Vasc. Biol..

[133] Combi Z., Potor L., Nagy P. (2023). Hydrogen sulfide as an anti-calcification stratagem in human aortic valve: altered biogenesis and mitochondrial metabolism of H2S lead to H2S deficiency in calcific aortic valve disease. Redox Biol..

[134] Yi H., Li X.H., Yi B. (2010). Identification of Rack1, EF-tu and rhodanese as aging-related proteins in human colonic epithelium by proteomic analysis. J. Proteome Res..

[135] Taniguchi E., Matsunami M., Kimura T. (2009). Rhodanese, but not cystathionine-γ-lyase, is associated with dextran sulfate sodium-evoked colitis in mice: a sign of impaired colonic sulfide detoxification?. Toxicology.

[136] De Preter V., Arijs I., Windey K. (2012). Decreased mucosal sulfide detoxification is related to an impaired butyrate oxidation in ulcerative colitis. Inflamm. Bowel Dis..

[137] Stummer N., Weghuber D., Feichtinger R.G. (2022). Hydrogen sulfide metabolizing enzymes in the intestinal mucosa in pediatric and adult inflammatory bowel disease. Antioxidants.

[138] Wang R.H., Chu Y.H., Lin K.T. (2021). The hidden role of hydrogen sulfide metabolism in cancer. Int. J. Mol. Sci..

[139] Ansar M., Thu L.T.A., Hung C.S. (2022). Promoter hypomethylation and overexpression of TSTD1 mediate poor treatment response in breast cancer. Front. Oncol..

[140] Ramasamy S., Singh S., Taniere P., Langman M.J.S., Eggo M.C. (2006). Sulfide-detoxifying enzymes in the human colon are decreased in cancer and upregulated in differentiation. Am. J. Physiol. Gastrointest. Liver Physiol..

[141] Kaczor-Kamińska M., Kaminski K., Wróbel M. (2021). The expression and activity of rhodanese, 3-mercaptopyruvate sulfurtransferase, cystathionine γ-lyase in the most frequently chosen cellular research models. Biomolecules.

[142] Al‐Dahmani Z.M., Hadian M., Ruiz‐Moreno A.J. (2023). Identification and characterization of a small molecule that activates thiosulfate sulfurtransferase and stimulates mitochondrial respiration. Protein Sci..

[143] Slade L., Deane C.S., Szewczyk N.J., Etheridge T., Whiteman M. (2024). Hydrogen sulfide supplementation as a potential treatment for primary mitochondrial diseases. Pharmacol. Res..

[144] Macabrey D., Joniová J., Gasser Q. (2022). Sodium thiosulfate, a source of hydrogen sulfide, stimulates endothelial cell proliferation and neovascularization. Front. Cardiovasc. Med..

[145] Frenay A.R.S., de Borst M.H., Bachtler M. (2016). Serum free sulfhydryl status is associated with patient and graft survival in renal transplant recipients. Free Radic. Biol. Med..

[146] Sakaguchi M., Marutani E., sook Shin H. (2014). Sodium thiosulfate attenuates acute lung injury in mice. Anesthesiology.

[147] Nguyen I.T.N., Klooster A., Minnion M. (2020). Sodium thiosulfate improves renal function and oxygenation in L-NNA–induced hypertension in rats. Kidney Int..

[148] Baskin S.I., Horowitz A.M., Nealley E.W. (1992). The antidotal action of sodium nitrite and sodium thiosulfate against cyanide poisoning. J. Clin. Pharmacol..

[149] Olson K.R., DeLeon E.R., Gao Y. (2013). Thiosulfate: a readily accessible source of hydrogen sulfide in oxygen sensing. Am. J. Physiol. Regul. Integr. Comp. Physiol..

[150] Iciek M., Bilska-Wilkosz A., Górny M., Sokołowska-Jeżewicz M., Kowalczyk-Pachel D. (2016). The effects of different garlic-derived allyl sulfides on anaerobic sulfur metabolism in the mouse kidney. Antioxidants.

[151] Macabrey D., Longchamp A., MacArthur M.R. (2022). Sodium thiosulfate acts as a hydrogen sulfide mimetic to prevent intimal hyperplasia via inhibition of tubulin polymerisation. EBioMedicine.

[152] Whiteman M., Le Trionnaire S., Chopra M., Fox B., Whatmore J. (2011). Emerging role of hydrogen sulfide in health and disease: critical appraisal of biomarkers and pharmacological tools. Clin. Sci..

[153] Bilska-Wilkosz A., Iciek M., Górny M., Kowalczyk-Pachel D. (2017). The role of hemoproteins: hemoglobin, myoglobin and neuroglobin in endogenous thiosulfate production processes. Int. J. Mol. Sci..

[154] Zhang M.Y., Dugbartey G.J., Juriasingani S., Sener A. (2021). Hydrogen sulfide metabolite, sodium thiosulfate: clinical applications and underlying molecular mechanisms. Int. J. Mol. Sci..

[155] Combi Z., Potor L., Nagy P. (2023). Hydrogen sulfide as an anti-calcification stratagem in human aortic valve: altered biogenesis and mitochondrial metabolism of H2S lead to H2S deficiency in calcific aortic valve disease. Redox Biol..

[156] Bronowicka-Adamska P., Kaczor-Kamińska M., Wróbel M., Bentke-Imiolek A. (2024). Differences in nonoxidative sulfur metabolism between normal human breast MCF-12A and adenocarcinoma MCF-7 cell lines. Anal. Biochem..

[157] Jurkowska H., Wróbel M., Jasek-Gajda E., Rydz L. (2022). Sulfurtransferases and cystathionine beta-synthase expression in different human leukemia cell lines. Biomolecules.

[158] Jurkowska H., Wróbel M. (2008). N-acetyl-L-cysteine as a source of sulfane sulfur in astrocytoma and astrocyte cultures: correlations with cell proliferation. Amino Acids.

[159] Ascenção K., Dilek N., Zuhra K., Módis K., Sato T., Szabo C. (2022). Sequential accumulation of ‘driver’ pathway mutations induces the upregulation of hydrogen-sulfide-producing enzymes in human colonic epithelial cell organoids. Antioxidants.

[160] Szlęzak D., Hutsch T., Ufnal M., Wróbel M. (2022). Heart and kidney H2S production is reduced in hypertensive and older rats. Biochimie.

[161] Revenko O., Pavlovskiy Y., Savytska M. (2021). Hydrogen sulfide prevents mesenteric adipose tissue damage, endothelial dysfunction, and redox imbalance from high fructose diet-induced injury in aged rats. Front. Pharmacol..

[162] Zheng A., Li H., Feng Z., Liu J. (2021). Integrative analyses reveal Tstd1 as a potential modulator of HDL cholesterol and mitochondrial function in mice. Cells.

[163] Nakajima T., Taki K., Wang B. (2008). Induction of rhodanese, a detoxification enzyme, in livers from mice after long-term irradiation with low-dose-rate gamma-rays. J. Radiat. Res..

[164] Pavlovskiy Y., Yashchenko A., Zayachkivska O. (2020). H2S donors reverse age-related gastric malfunction impaired due to fructose-induced injury via CBS, CSE, and TST expression. Front. Pharmacol..

[165] Szlęzak D., Bronowicka-Adamska P., Hutsch T., Ufnal M., Wróbel M. (2021). Hypertension and aging affect liver sulfur metabolism in rats. Cells.

[166] Iciek M., Górny M., Kotańska M., Bilska-Wilkosz A., Kaczor-Kamińska M., Zagajewski J. (2023). Yohimbine alleviates oxidative stress and suppresses aerobic cysteine metabolism elevated in the rat liver of high-fat diet-fed rats. Molecules.

[167] Wang D., Liu Y., Zong X. (2023). Sodium thiosulfate ameliorates atopic dermatitis via inhibiting the activation of NLRP3 inflammasome. Biochem. Biophys. Res. Commun..

[168] Wolfschmitt E.M., Hogg M., Vogt J.A. (2023). The effect of sodium thiosulfate on immune cell metabolism during porcine hemorrhage and resuscitation. Front. Immunol..

[169] Wang D., Li S., Chen Y. (2023). Sodium thiosulfate inhibits epithelial-mesenchymal transition in melanoma via regulating the Wnt/β-catenin signaling pathway. J. Dermatol. Sci..

[170] Macabrey D., Longchamp A., MacArthur M.R. (2022). Sodium thiosulfate acts as a hydrogen sulfide mimetic to prevent intimal hyperplasia via inhibition of tubulin polymerisation. EBioMedicine.

[171] Nelson P., Dugbartey G.J., McFarlane L. (2024). Effect of sodium thiosulfate pre-treatment on renal ischemia-reperfusion injury in kidney transplantation. Int. J. Mol. Sci..

[172] Wang D., Liu Y., Zong X. (2023). Sodium thiosulfate ameliorates atopic dermatitis via inhibiting the activation of NLRP3 inflammasome. Biochem. Biophys. Res. Commun..

[173] Press A.T., Ungelenk L., Medyukhina A. (2023). Sodium thiosulfate refuels the hepatic antioxidant pool reducing ischemia-reperfusion-induced liver injury. Free Radic. Biol. Med..

[174] Shekari M., Gortany N.K., Khalilzadeh M. (2022). Cardioprotective effects of sodium thiosulfate against doxorubicin-induced cardiotoxicity in male rats. BMC Pharmacol Toxicol.

[175] Hsu C.N., Hou C.Y., Chang-Chien G.P., Lin S., Yang H.W., Tain Y.L. (2022). Sodium thiosulfate improves hypertension in rats with adenine-induced chronic kidney disease. Antioxidants.

[176] Gonzales J., Sabatini S. (1989). Cyanide poisoning: pathophysiology and current approaches to therapy. Int. J. Artif. Organs.

[177] Brock P.R., Maibach R., Childs M. (2018). Sodium thiosulfate for protection from cisplatin-induced hearing loss. N. Engl. J. Med..

[178] Tsang R.Y., Al-Fayea T., Au H.J. (2009). Cisplatin overdose. Drug Saf..

[179] Abeck F., Hansen I., Rünger A., Booken N., Schneider S.W. (2024). Successful treatment of non‐uremic calciphylaxis with combination therapy with sodium thiosulfate, iloprost, and heparin. Int. J. Dermatol..

[180] Ning M.S., Dahir K.M., Castellanos E.H., McGirt L.Y. (2013). Sodium thiosulfate in the treatment of non‐uremic calciphylaxis. J. Dermatol..

[181] Hunt G.M., Ryder H.F. (2018). Metabolic acidosis after sodium thiosulfate infusion and the role of hydrogen sulfide. Clin Case Rep.

[182] Sun Y., Huang Y., Zhang R. (2015). Hydrogen sulfide upregulates KATP channel expression in vascular smooth muscle cells of spontaneously hypertensive rats. J. Mol. Med..

